# siRNA Silencing of Proteasome Maturation Protein (*POMP*) Activates the Unfolded Protein Response and Constitutes a Model for KLICK Genodermatosis

**DOI:** 10.1371/journal.pone.0029471

**Published:** 2012-01-03

**Authors:** Johanna Dahlqvist, Hans Törmä, Jitendra Badhai, Niklas Dahl

**Affiliations:** 1 Science for Life Laboratory, Department of Immunology, Genetics and Pathology, Uppsala University, Uppsala, Sweden; 2 Department of Medical Sciences, Uppsala University and University Hospital, Uppsala, Sweden; University of Birmingham, United Kingdom

## Abstract

Keratosis linearis with ichthyosis congenita and keratoderma (KLICK) is an autosomal recessive skin disorder associated with a single-nucleotide deletion in the 5′untranslated region of the proteasome maturation protein (*POMP*) gene. The deletion causes a relative switch in transcription start sites for *POMP*, predicted to decrease levels of POMP protein in terminally differentiated keratinocytes. To investigate the pathophysiology behind KLICK we created an *in vitro* model of the disease using siRNA silencing of *POMP* in epidermal air-liquid cultures. Immunohistochemical analysis of the tissue constructs revealed aberrant staining of POMP, proteasome subunits and the skin differentiation marker filaggrin when compared to control tissue constructs. The staining patterns of *POMP* siRNA tissue constructs showed strong resemblance to those observed in skin biopsies from KLICK patients. Western blot analysis of lysates from the organotypic tissue constructs revealed an aberrant processing of profilaggrin to filaggrin in samples transfected with siRNA against *POMP*. Knock-down of *POMP* expression in regular cell cultures resulted in decreased amounts of proteasome subunits. Prolonged silencing of *POMP* in cultured cells induced C/EBP homologous protein (CHOP) expression consistent with an activation of the unfolded protein response and increased endoplasmic reticulum (ER) stress. The combined results indicate that KLICK is caused by reduced levels of POMP, leading to proteasome insufficiency in differentiating keratinocytes. Proteasome insufficiency disturbs terminal epidermal differentiation, presumably by increased ER stress, and leads to perturbed processing of profilaggrin. Our findings underline a critical role for the proteasome in human epidermal differentiation.

## Introduction

KLICK genodermatosis (MIM #601952) is an autosomal recessive skin disorder characterized by ichthyosis, hyperkeratotic plaques, palmoplantar hyperkeratosis, circular constrictions around fingers and numerous papules in a linear pattern in armfolds and on wrists [1], [2], [3]. A single-nucleotide deletion in the 5′ untranslated region (UTR; c.-95DelC) of the *POMP* gene was recently identified in a homozygous state in patients with KLICK [4]. The mutation is associated with a switch in transcription start sites (TSS's) used for *POMP*, resulting in a 29-fold increase in the proportion of transcripts with long 5′UTR's in differentiated keratinocytes. Immunohistochemical analysis of skin biopsies from KLICK patients display altered expression of POMP, proteasome subunits and the epidermal differentiation marker filaggrin. Furthermore, differentiated epidermal layers of patient skin biopsies show increased staining of the unfolded protein response (UPR) protein CHOP, suggesting that ER stress is a pathophysiological mechanism in KLICK [4]. However, molecular and cellular studies of keratinocytes with the *POMP* 5′UTR mutation have been hampered due to difficulties in obtaining terminally differentiated keratinocytes *in vitro*.

The proteasome is a large protein complex, step-wise assembled by α and β subunits forming hemiproteasomes which dimerize to form a complete proteasome [5], [6]. POMP is essential for the incorporation of β subunits, the dimerization of hemiproteasomes and consequently for normal proteasome function [7], [8]. However, the direct impact of a deficient POMP function on proteasome α and β subunit levels has not been thoroughly investigated. Under physiological conditions misfolded and folding-incompetent proteins in the ER are retrotranslocated to the cytoplasm, polyubiquitinated and degraded by the proteasome [9]. It is known that inhibition of the proteasome impedes ER function and induces ER stress, but whether POMP insufficiency has the same consequences is unknown [10], [11]. ER stress activates the UPR, a cellular response to restore ER homeostasis. As proteins accumulate in the ER, the chaperone BiP dissociates from ER transmembrane receptors, which then induce different UPR pathways aiming to attenuate protein translation, induce expression of ER and UPR factors and increase protein degradation [12], [13], [14], [15].

In this study we present additional evidence for an association between decreased amounts of POMP, increased ER stress and KLICK genodermatosis. We show that *in vitro* knock-down of *POMP* expression in organotypic epidermal tissue constructs mimics the immunohistochemical phenotype of KLICK epidermis in terms of aberrant POMP, filaggrin and proteasome subunit distribution. Silencing of *POMP* expression in cell culture results in decreased amounts of proteasome subunits and increased ER stress. These results underline the importance of proteasomes in epidermal differentiation and link ER stress to aberrant epidermal differentiation in KLICK genodermatosis.

## Results

### Long mutant 5′ UTR shows a strong tendency for reducing POMP translation efficiency

**Figure 1:**
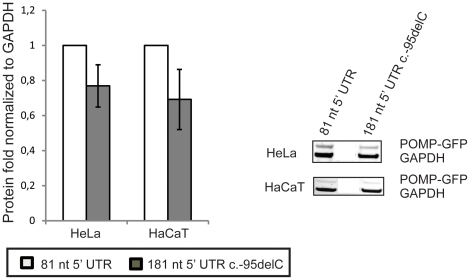
Effect of different *POMP* 5′ UTR's on translation efficiency. Fusion protein levels of POMP-GFP were measured by western blot analysis of cells transfected with POMP-GFP constructs (right panel). Two different variants of POMP cDNA were analyzed, differing in their 5′ UTR: one clone had an 81-nt-5′ UTR, wild type, and one clone had a 181-nt-5′ UTR with mutation c.-95delC. Fusion protein levels of 181-nt-5′ UTR c.-95delC constructs are plotted as relative expression from the 81-nt-5′ UTR construct. Results are based on three separate experiments and are presented as mean values +/− standard deviation (SD). Fusion protein levels were related to GFP levels of co-transfected empty vectors and to GAPDH as internal control.

We have previously shown in overexpression studies that a long *POMP* wild type 5′ UTR reduces POMP-GFP fusion protein levels compared to short 5′ UTR POMP constructs [4]. To investigate the effects of a long 5′ UTR including the KLICK-associated variant c.-95delC in comparison to a short wild type 5′ UTR on POMP protein expression we overexpressed two constructs containing full-length *POMP* cDNA with different 5′ UTR's; one with an 81-nt-5′ UTR, wild type, and one with a 181-nt-5′ UTR including c.-95DelC. The constructs were fused with GFP and transiently expressed in HeLa and HaCaT cells. Western blot analysis revealed a 23% reduction in POMP-GFP expression from the mutant 181-nt-5′UTR construct compared to the wild type 81-nt-5′UTR construct in HeLa cells (One-Sample (OS) t-test p = 0.0801, Mann-Whitney (MW) p = 0.0636) and a 31% reduction in HaCaT cells (OS t-test p = 0.0894, MW p = 0.0636; Fig. 1).

### *POMP* siRNA transfected epidermal tissue constructs mimic KLICK epidermis

**Figure 2:**
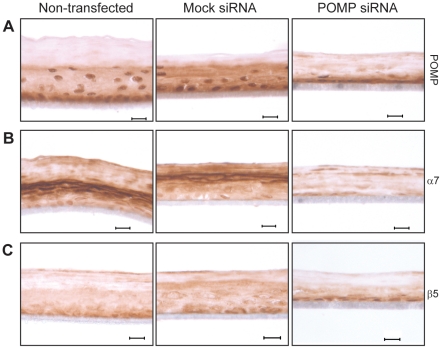
Proteasome subunit analysis of keratinocyte air-liquid cultures transfected with siRNA against *POMP*. Epidermal tissue constructs were established without transfection and after transfection with *POMP* siRNA or mock siRNA. Sections of the mature tissue constructs were immunostained with (**A**) anti-POMP, (**B**) anti-α7 proteasome subunit and (**C**) anti-β5 proteasome subunit antibodies. *POMP* siRNA transfected tissue cultures showed *POMP* mRNA levels reduced to 46% of the levels in mock transfected cells. Results are based on three separate experiments. Bar: 20 µm.

Next, we investigated whether siRNA silencing of *POMP* expression in keratinocyte air-liquid cultures can function as a model of KLICK *in vitro*. Cells transfected with *POMP* siRNA or mock siRNA as well as non-transfected cells were cultured for 10–12 days on polycarbonate inserts. Subsequent mRNA analysis showed an average reduction in *POMP* transcript levels of 54% and 53% in *POMP* siRNA transfected tissue constructs compared to mock siRNA transfected and non-transfected tissue constructs, respectively (data not shown). Sections of the tissue constructs were analyzed together with skin biopsies derived from healthy controls and KLICK patients [4] by immunohistochemical staining of POMP and proteasome subunits α7 and β5. *POMP*-silenced organotypic epidermis showed a weak staining of POMP and a weak and patchy staining of α7 proteasome subunits compared to non-transfected and mock-transfected tissue cultures (Fig. 2 A–B), whilst the staining of β5 proteasome subunits was similar between all tissue constructs (Fig. 2 C). The staining patterns of proteasome subunits in *POMP*-silenced organotypic epidermis are in almost complete agreement with those observed in skin biopsies from KLICK patients using the same antibodies [4].

### POMP deficiency disrupts epidermal profilaggrin processing

**Figure 3:**
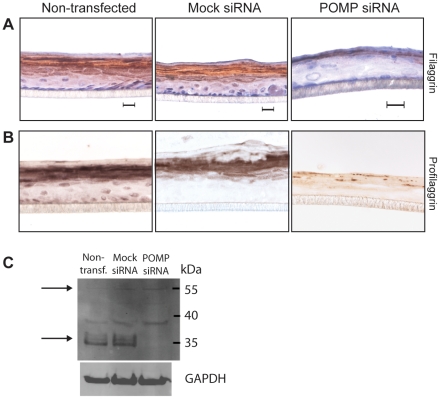
Analysis of profilaggrin processing in keratinocyte air-liquid cultures transfected with siRNA against *POMP*. Epidermal tissue constructs were established without transfection and after transfection with mock siRNA or *POMP* siRNA, respectively. Sections of the mature tissue constructs were immunostained with (**A**) anti-filaggrin (Novocastra) and (**B**) anti-profilaggrin antibodies (Abcam). Magnification: ×63. (**C**) Lysates was prepared from the three epidermal tissue constructs and analyzed using western blot analysis with immunodetection of the N-terminus of profilaggrin. Predicted profilaggrin cleavage products (N-terminal domain (32 kDa) and N-terminal domain with truncated filaggrin unit (55–57 kDa)) are indicated by arrows, respectively (upper panel). GAPDH was used as internal control. Results are based on three separate experiments. Non-transf. = non-transfected tissue construct; mock siRNA = mock siRNA transfected tissue construct; POMP siRNA = *POMP* siRNA transfected tissue construct.

Next, we stained sections from the epidermal tissue constructs with antibodies against filaggrin. We observed a weak and patchy staining of the cornified cell layer of *POMP*-silenced organotypic epidermis, compared to control sections (Fig. 3 A). Again, these staining patterns are in agreement with those of skin from KLICK patients and healthy controls, respectively [4]. Filaggrin derives from the precursor protein profilaggrin, which consists of an N-terminal domain, followed by a truncated filaggrin unit, 10–12 complete filaggrin units and a C-terminal domain. Profilaggrin is proteolytically cleaved into its subunits in differentiated epidermis [16]. To investigate whether the expression and proteolytic processing of profilaggrin is affected by POMP deficiency we stained skin sections from healthy controls and KLICK patients and sections from the epidermal tissue constructs with antibodies against the N-terminal domain of profilaggrin. The immunostaining of healthy skin was confined to the outermost cells of the granular cell layer and the innermost cells of the cornified layer (Fig. S1 A) whereas sections from KLICK patients showed an irregular staining of the same cell layers and, additionally, a patchy staining of the entire cornified layer with preserved granules of profilaggrin in the outermost cells (Fig. S1 B–C). Sections from *POMP*-silenced epidermal tissue constructs showed a reduced immunostaining of the granular cell layer when compared to the non-transfected and mock siRNA transfected epidermal tissue constructs (Fig. 3 B). Similar to what was observed in skin from KLICK patients the *POMP* siRNA treated tissue constructs also showed a retention of profilaggrin granules in the cornified cell layer (Fig. 3 B). To verify that POMP deficiency perturbs profilaggrin processing, lysates from the organotypic constructs were analyzed by western blot and immunodetection of the N-terminal domain of profilaggrin. Interestingly, the cleaved N-terminal domain (32–33 kDa) was not detected in lysates from *POMP* siRNA transfected constructs but clearly observed in lysates from non-transfected and mock siRNA transfected tissue constructs (Fig. 3 C).

### Silencing of *POMP* expression in cultured cells causes a decrease in proteasome subunit levels

**Figure 4:**
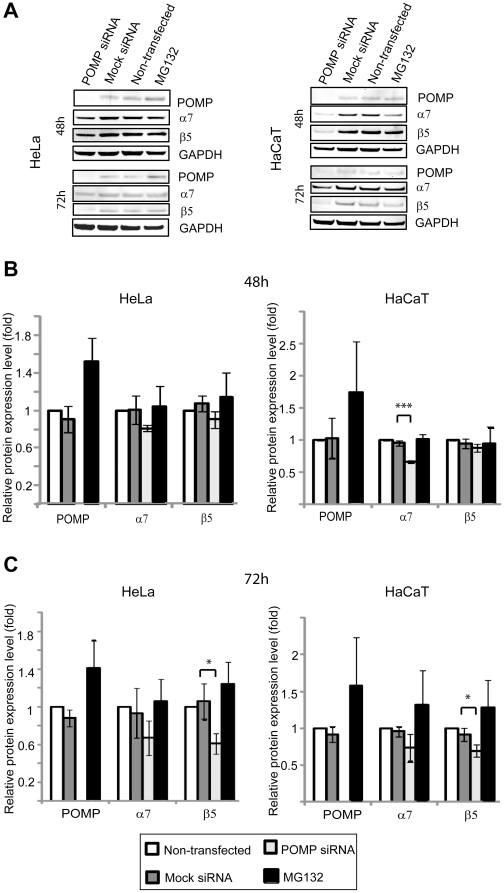
Proteasome subunit analysis of *POMP* siRNA transfected cell lines. Crude lysates were prepared from HeLa and HaCaT cells transfected with *POMP* siRNA or mock siRNA and non-transfected cells (+/−1 µM MG132). Western blot analyses were run for POMP and proteasome subunits α7 and β5 at 48 h (**A**, **B**) and 72 h (**A**, **C**) post transfection. Results are based on three separate experiments and are presented as mean values +/− SD. GAPDH was used as internal control. Protein levels of siRNA transfected and MG132 treated cells were normalized against non-transfected cells. Differences between *POMP* siRNA and mock siRNA transfected cells were analyzed using Student's t-test; * = p<0.05, *** = p<0.001.

We next wanted to elucidate the direct effects of absent POMP on α and β proteasome subunits, by silencing *POMP* expression in HeLa and HaCaT cells. Cells transfected with mock siRNA, non-transfected cells and non-transfected cells treated with proteasome inhibitor MG132 were used for comparison. *POMP* siRNA reduced *POMP* mRNA levels to 8.0% (HeLa) and 5.6% (HaCaT) of levels in mock transfected cells at 48 h after transfection and to 5.7% (HeLa) and 6.9% (HaCaT) at 72 h after transfection (Fig. S2 A–B). There were no measurable levels of POMP protein after 48 h or 72 h in either of the two *POMP* siRNA transfected cell types (Fig. 4 A–C). mRNA and protein levels of proteasome subunits α7 and β5 were first measured at 48 h after transfection. Decreased levels of α7 protein were observed in HaCaT cells (t-test p = 0.000386, MW p = 0.1) and a tendency for a decrease in α7 and β5 levels was seen in HeLa cells (α7 t-test p = 0.0860, MW p = 0.1; β5 t-test p = 0.0696, MW p = 0.2; Fig. 4 A–B), whilst the mRNA levels were mainly unaffected (Fig. S2 A). At 72 h after transfection β5 protein levels were reduced in both cell types (HeLa t-test p = 0.0220, MW p = 0.1; HaCaT t-test p = 0.0400, MW p = 0.1; Fig. 4 A, C) whereas mRNA levels of both *α7* and *β5* were increased in both cell types (HeLa α7 t-test p = 0.00539, MW p = 0.1, β5 t-test p = 0.0243, MW p = 0.1; HaCaT α7 t-test p = 0.0444, MW p = 0.1, β5 t-test p = 0.0265, MW p = 0.1; Fig. S2 B) compared to mock transfected cells. MG132 treated cells showed an increase in *α7*, *β5* and *POMP* mRNA and in POMP protein levels, as described previously [17].

### Silencing of *POMP* expression in cultured cells activates the UPR

**Figure 5:**
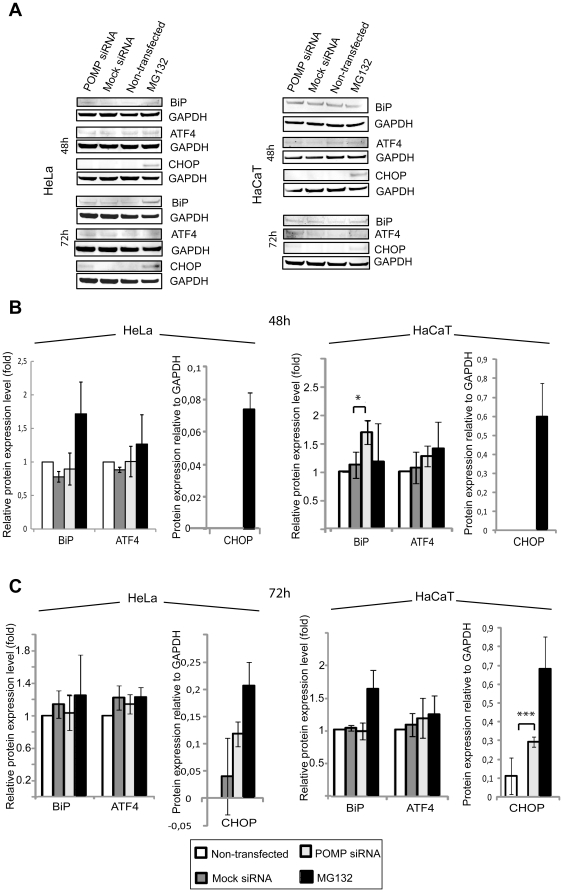
UPR protein analysis of *POMP* siRNA transfected cell lines. Crude lysates were prepared from HeLa and HaCaT cells transfected with *POMP* siRNA or mock siRNA and non-transfected cells (+/−1 µM MG132). Western blot analyses were run for UPR markers BiP, ATF4 and CHOP at 48 h (**A**, **B**) and 72 h (**A**, **C**) post transfection. Results are based on three separate experiments and are presented as mean values +/− SD. GAPDH was used as internal control. BiP and ATF4 protein levels of siRNA transfected and MG132 treated cells were normalized against non-transfected cells. Differences between *POMP* siRNA and mock siRNA transfected cells were analyzed using Student's t-test; * = p<0.05, *** = p<0.001.

Proteasome inhibitors are known to induce the UPR [18], [19] and lead us to investigate whether a similar effect could be observed in cells depleted of POMP. mRNA and protein levels of ER chaperone BiP and UPR transcription factors ATF4 and CHOP were analyzed in cells transfected with siRNA against *POMP*. At 48 h after transfection an increase in BiP protein levels was observed in HaCaT (t-test p = 0.0352, MW p = 0.1) but not in HeLa cells (Fig. 5 A–B). None of the two cell types showed any significant changes in ATF4 levels or measurable levels of CHOP proteins and there were no changes in *BiP*, *ATF4* or *CHOP* mRNA levels (Fig. S2 C). At 72 h after transfection of POMP siRNA CHOP protein was expressed in both HeLa and HaCaT cells (HeLa t-test p = 0.142, MW p = 0.631, HaCaT t-test p = 4.89*10^−5^, MW p = 0.0636) in three out of three experiments, while there were no changes in BiP or ATF4 levels (Fig. 5 A–C). A very weak detection of CHOP was observed in one western blot experiment for mock transfected and non-transfected cells respectively. *CHOP* mRNA levels were increased in HeLa cells (t-test p = 0.0275, MW p = 0.1) and *BiP* mRNA was increased in HaCaT cells (t-test p = 0.0359, MW 0.1; Fig. S2 D). MG132 treated cells showed increased mRNA levels for all three UPR markers in accordance with previous studies [18], [19] while protein measurements revealed an increase in CHOP.

## Discussion

Terminal differentiation of human epidermis requires a network of cooperating events, involving complex and spatial assembly and degradation of proteins and lipids [20]. We have previously reported on aberrant epidermal differentiation in patients with KLICK genodermatosis [4]. Affected individuals are homozygous for a single-nucleotide deletion in the 5′ UTR of the *POMP* gene. The mutation is associated with a transcriptional switch towards *POMP* transcripts with long 5′ UTRs, particularly in differentiated keratinocytes where long 5′ UTR transcripts constitute 83% of all POMP transcripts in patients but only 2.6% in controls [4]. Skin biopsies from KLICK patients show abnormal distribution of proteasomes as well as an indication of increased UPR. We present herein evidence for a disturbed keratinocyte differentiation due to POMP insufficiency which emphasizes the importance of the proteasome system in terminal epidermal differentiation. In addition, POMP depleted cells show an activated UPR, providing further support for the hypothesis of ER stress as an important pathophysiological mechanism in KLICK.

Transcripts with long 5′ UTR's are generally associated with reduced translation rate [21]. We were, despite this, unable to detect decreased POMP protein levels in cultured keratinocytes from KLICK patients in our previous study [4]. In the present study we hypothesized that the effect of an increased proportion of *POMP* transcripts with long 5′ UTR on translational efficiency is detectable only in terminally differentiated keratinocytes. To clarify the role of 5′ UTR length and the single-nucleotide deletion on POMP translation we over-expressed *POMP* constructs with different length of the 5′ UTR, in two different cell types. We found that constructs expressing a long 5′ UTR (181 nt) with the KLICK mutation produce 23–31% less protein than shorter 5′ UTR (81 nt) constructs. These findings indicate that KLICK patients, predominantly expressing long 5′UTR *POMP* transcripts in differentiated keratinocytes, have a decreased POMP translation rate in differentiated epidermis.

We next wanted to ensure that reduced POMP protein levels cause the abnormal epidermal differentiation seen in KLICK skin [4], using epidermal tissue constructs as an *in vitro* model of human interfollicular epidermis [22]. The constructs mimic the stratification of cells into basal, spinous, granular and cornified layers, but the thickness of different layers may differ from epidermis due to the humidity and culture time of the air-liquid systems, as well as the lack of mechanical stress. We established epidermal tissue constructs in air-liquid culture systems pre-transfected with siRNA against *POMP*. Interestingly, after 10–12 days we observed an aberrant staining of POMP and proteasome subunit α7 in siRNA transfected tissue constructs where the patchy staining of α7 resembles that of KLICK skin biopsies. This indicates that the deviations observed in KLICK skin are specific effects of POMP insufficiency and a disturbed proteasome function. Moreover, the incoherent staining of profilaggrin and filaggrin in KLICK skin was apparent also in *POMP* siRNA transfected tissue constructs. Although the expression of profilaggrin in tissue constructs seems to be reduced when compared to skin of KLICK patients, we observe a clear effect on profilaggrin processing associated with POMP deficiency. Western blot analysis of the N-terminus of profilaggrin revealed an aberrant pattern with absence of the N-terminal cleavage product of profilaggrin comprising the profilaggrin A and B domains [16], [23], [24] in *POMP*-silenced tissue constructs. In combination, these findings indicate that the proteasome is involved in profilaggrin processing and that KLICK is associated with a disturbed epidermal expression of filaggrin.

From our results we concluded that siRNA silencing of *POMP* models KLICK syndrome *in vitro*. We then studied the effects of knock-down of *POMP* in regular cell cultures. It has previously been shown that silencing of *POMP* expression by siRNA results in abolished incorporation of β5 and β5i subunits into proteasomes and hence a decreased amount of mature proteasomes [6], [7]. In support of this we observed decreased amounts of α7 proteasome subunits in HaCaT cells at 48 h post siRNA transfection and decreased levels of β5 subunits in both HaCaT and HeLa cells at 72 h post transfection. The results imply that reduced POMP levels lead to degradation of free subunits, with cell type specific differences in response time between α7 and β5 subunits. The reduction in amounts of subunit proteins was associated with a compensatory transcriptional up-regulation of α7 and β5 subunit genes. The effect of *POMP* knock-down on proteasome subunits explains the weaker expression of α7 and β5 in KLICK epidermis. Interestingly, there was no decrease in α7 and β5 subunits in cells treated with the proteasome inhibitor MG132, indicating that the subunits remain stable if proteasomes are inhibited at a mature stage.

Proteasome degradation and the UPR are tightly regulated and connected systems of critical importance for the cell. The systems relieve the ER from protein overload, e.g. related to protein misfolding, high protein production or defect protein degradation [15], [18]. Keratinocytes of the granular epidermal layer are highly protein secreting cells with a physiological ER stress and active UPR [25], [26]. This makes keratinocytes vulnerable for a compromised ER function as shown for UVB exposed epidermis and in erythrokeratoderma variabilis [27], [28]. We analyzed ER chaperone BiP and UPR markers CHOP and ATF4 in cells depleted of POMP after siRNA transfection. We found a significant up-regulation of CHOP after prolonged (72 h) knock-down of *POMP* in both HeLa and HaCaT cells. CHOP is generally not expressed under physiological conditions, but is activated at later stages of the UPR [13] consistent with the expression in our experiments. Furthermore, *POMP* knock-down caused a slight increase in the ER chaperone BiP in keratinocyte derived HaCaT cells, but not in HeLa cells, supporting tissue specific sensitivity to ER stress, as suggested previously [18]. No changes were seen in ATF4 levels for either of the cell types. Levels of BiP and ATF4 increase early in the UPR [18], [19] and we cannot exclude that marked changes in levels of these proteins take place before 48 h.

Taken together, we show that the *POMP* 5′ UTR mutation associated with KLICK genodermatosis results in reduced POMP expression, which in turn causes reduced levels of proteasome subunits and filaggrin as well as increased ER stress. Our results are consistent with different possible mechanisms contributing to perturbed epidermal differentiation in KLICK genodermatosis: Firstly, the cleavage products of profilaggrin have been shown to be indispensable in the formation of the epidermal water barrier [16], [29] and we show that the proteasome is essential for adequate profilaggrin processing. Secondly, the proteasome is a known regulator of key proteins in several cellular processes and has, for instance, been shown to control the levels of retinoic acid receptor gamma, which is important for transcriptional regulation in epidermis [30]. Thirdly, aggregation of unfolded proteins hampers normal cellular processes and the UPR has been shown to directly affect the expression of genes involved in epidermal differentiation [25], [31].

Further studies are required to clarify the exact role of the proteasome in epidermal differentiation and the molecular effects of increased ER stress in epidermis. Increased understanding of ER stress involvement in KLICK genodermatosis and epidermal differentiation may open up for new therapeutic strategies of potential importance for a large group of skin disorders.

## Materials and Methods

### Ethics statement

This project was approved by the local Research Ethics Committee Uppsala. The use of human samples is conducted according to the principles expressed in the Declaration of Helsinki.

### Cell culture

HeLa cells [32] were cultured in RPMI 1640 with 10% Fetal bovine serum (FBS), 100 U/ml Penicillin-Streptomycin (PEST) and 2 mM L-glutamine (all GIBCO/Invitrogen, Paisley, UK). The HaCaT keratinocyte cell line [33] was cultured in calcium-free DMEM (Invitrogen) with 10% chelexed FBS, 100 U/ml PEST, 2 mM L-glutamine and supplement of 0.03 mM CaCl_2_. All cells were cultured in 37 degrees Celsius with 5% CO_2_ in a humid environment.

For air-liquid cultures we used second to third passage epidermal keratinocytes expanded in EpiLife™ medium (Invitrogen) that were seeded the day after siRNA transfection on Millicell-PCF inserts (pore size 0.4 µm; Millipore AB, Solna, Sweden) at a density of 3×10^5^ cells/insert in 1.5 mM CaCl_2_ supplemented medium. The keratinocytes were cultured as described previously [16] for 10–12 days after which punch biopsies were taken from the tissue constructs for analysis by immunohistochemistry and quantitative real-time PCR.

### Cloning and transfection of reporter constructs

cDNA clones corresponding to two variants of full length *POMP* cDNA were cloned into a fluorescent reporter vector (pAcGFP1-N1; Clontech, Saint-Germain-en-Laye, France) to produce POMP-GFP fusion proteins, as described previously [4]. The two clone variants differ in their 5′ UTR: one clone corresponds to wild type *POMP* transcripts with an 81-nucleotide 5′ UTR (81-wt-5′UTR) and one clone is associated with KLICK genodermatosis having a 181-nucleotide 5′ UTR with a cytosine deletion (c.-95DelC). All plasmids were verified by sequencing. HeLa and HaCaT cells were transfected with either of the POMP-GFP vectors together with empty vector using Lipofectamine 2000 (Invitrogen) according to the manufacturer's recommendations. Cells were harvested after 48 hours by trypsinization for protein preparation. Statistical calculations were performed with results from three experiment replicates using both One-sample t-test (OS; to give an indication of significance of differences between samples) and Mann-Whitney's test (MW; due to the low number of observations), using R software.

### Gene silencing

For silencing of *POMP* expression Invitrogen Stealth Select RNAi™ siRNA (oligo ID: HSS147436, HSS147437) was used. Cultured keratinocytes, HeLa and HaCaT cells were transfected with *POMP* siRNA using Lipofectamine RNAiMax (Invitrogen) according to the protocol provided by the manufacturer. HeLa and HaCaT cells were harvested at 48 h and 72 h after transfection for RNA and protein preparation. The two siRNA oligos worked equally well with a 92.0–94.4% reduction of POMP expression in HeLa and HaCaT experiments. Cells transfected with negative control (mock) siRNA (Stealth RNAi™ siRNA Negative Control LO GC, HI GC; Invitrogen), non-transfected cells and non-transfected cells treated with 1 µM proteasome inhibitor MG132 (Sigma Aldrich, St Louis, MS, US) for 20 h were used as negative and positive controls respectively. mRNA and protein analyses of *POMP* siRNA and mock siRNA transfected samples were performed with results from three separate experiments using Student's t-test and MW. Air-liquid cultures were set up in triplicates in three separate experiments and mock siRNA transfected and non-transfected cells were used as controls.

### Western blot analysis

HeLa and HaCaT cells were harvested by trypsinization and were after centrifugation resuspended in RIPA buffer (50 mM Tris–HCl pH 7.5, 150 mM NaCl, 1% Triton X-100, 1% sodium deoxycholate and 0.1% SDS; supplemented with MG132 proteasome inhibitor, phosphatase inhibitor cocktail 1, 0.1 mM sodium vanadate and protease inhibitor cocktail (all from Sigma Aldrich)) and kept at +4 degrees Celsius for 1 hour for protein preparation. The cell suspension was centrifuged for 10 min at +4 degrees Celsius at 13000 rpm to remove cell debris and the lysate was stored at −70 degrees Celsius until use [34]. Lysates were separated on Bis-Tris SDS-PAGE gels (Invitrogen), transferred to PVDF membranes (Millipore) and incubated with primary and secondary antibodies as described previously [34]. Secondary antibodies were conjugated with Alexa Fluor 680 (anti-mouse; Molecular probes/Invitrogen) or IRDye 800 (anti-rabbit; Li-Cor Bioscience, Cambridge, UK) for visualization with Odyssey infrared imaging system® (Li-Cor Bioscience). Immunodetection of target proteins was performed with anti-POMP, anti-α7 proteasome subunit, anti-β5 proteasome subunit (all BIOMOL, Hamburg, Germany), anti-CHOP (Cell Signaling technologies, Danvers, MA, US; Santa Cruz, Santa Cruz, CA, US), anti-BiP (Santa Cruz) and anti-ATF4 (Abcam, Cambridge, UK) antibodies for the siRNA experiments and anti-GFP (Clontech) antibody for the construct experiments. GADPH (Abcam) was used as internal control for all experiments and POMP-GFP fusion protein levels were adjusted for transfection efficiency by normalization against levels of co-transfected empty vector (pAcGFP1-N1). Western blot experiments for detection of profilaggrin degradation products in epidermal tissue constructs were performed similarly with tissue constructs lysed in RIPA buffer and antigens detected using anti-filaggrin antibodies (Abcam; ab81468 ) targeting the profilaggrin N-terminal domain.

### Quantitative real-time PCR

Total RNA was extracted from cultured HeLa and HaCaT cells and epidermal tissue constructs using Trizol (Invitrogen). The RNA was treated with DNase I (Sigma Aldrich) and reverse transcribed with RevertAid™ H Minus First Strand cDNA Synthesis Kit (Fermentas, Helsingborg, Sweden). Quantitative real-time PCR (qPCR) was run using Platinum SYBR Green qPCR supermix-UGD kit (Invitrogen) as described previously [35], with cDNA primers ordered from Sigma Aldrich (Table 1) and target gene expression levels normalized to beta actin levels.

**Table 1:** Primer sequences for qPCR analysis after siRNA transfection.

Primer	Sequence
POMP Forward	AAGTGAGCGGCGGGGTCGACT
POMP Reverse	CCTTTCCGAAGAAGATCATG
Alpha 7 Forward	TCTACAGTGCTGTTAGACCT
Alpha 7 Reverse	TATTTCCGTCTTTGCAGCTT
Beta 5 Forward	TGTAGCAGCTGCCTCCAAAC
Beta 5 Reverse	CCCTGAAATCCGGTTCCCTT
BiP Forward	CGGCCGCACGTGGA
BiP Reverse	CAACCACCTTGAACGGCAA
ATF4 Forward	CATGGGTTCTCCAGCGACAAG
ATF4 Reverse	TTGGAGGGACTGACCAACCC
CHOP Forward	TGCAGATTCACCATTCGGTC
CHOP Reverse	AGGAAATCGAGCGCCTGAC

### Immunohistochemistry

Epidermal tissue contructs were cut into 6 µm sections for protein visualization using primary antibodies against POMP (Abcam), α7 proteasome subunit, β5 proteasome subunit (both BIOMOL),filaggrin (Novocastra, Kista, Sweden) and profilaggrin (Abcam; targeting the profilaggrin N-terminal domain). The same antibody was used for immunohistochemical detection of profilaggrin on sections from skin biopsies from four healthy controls and three KLICK patients. Sections were fixed in 100% ice cold acetone and endogenous peroxidase activity was blocked by Peroxidazed 1 (Biocare Medical, Concord, CA, US). After reaction with Background Sniper (Biocare Medical) and incubation with primary antibodies, sections were incubated with biotinylated secondary antibodies (Vector Laboratories, Burlingame, CA, US). Thereafter the skin sections were incubated with avidin-biotin complex (Vector Laboratories) or Mach 3 Rabbit HRP polymer Detection Kit (Biocare Medical) for β5 subunit and peroxidase reactions were developed with DAB (Vector Laboratories). Filaggrin stained sections were counterstained with hematoxylin. Image analysis was performed with Leica DLMB microscope and Leica QWin software.

## Supporting Information

Figure S1 **Immunohistochemical detection of profilaggrin in human skin.** Epidermal sections from an healthy control (**A**) and patients with KLICK syndrome (**B**, **C**) were stained with antibodies against profilaggrin (Abcam). (**C**) Magnification of the cornified cell layer of patient epidermis. Bar: 50 µm.
(PDF)

Figure S2 **mRNA analysis of** ***POMP*** **siRNA transfected cell lines.** *POMP* was silenced in HeLa and HaCaT cells by siRNA transfection and cells transfected with mock siRNA and cells without transfection (+/−1 µM MG132) were used for comparison. mRNA levels of *POMP*, *α7*, *β5* (**A–B**), *BiP*, *ATF4* and *CHOP* (**C–D**) were analyzed by qPCR at 48 h (**A**, **C**) and 72 h (**B**, **D**) post transfection. Beta actin was used as internal control. Differences between *POMP* siRNA and mock siRNA transfected cells were analyzed using Student's t-test; * = p<0.05, *** = p<0.001.
(PDF)
