# Supplementary figures and images for: siRNA Silencing of Proteasome Maturation Protein (POMP) Activates the Unfolded Protein Response and Constitutes a Model for KLICK Genodermatosis
Source: PLoS One. 2012 Jan 3;7(1):e29471. doi: 10.1371/journal.pone.0029471 (PMC3250448; doi:10.1371/journal.pone.0029471)

Supporting information:  
Figure S1.

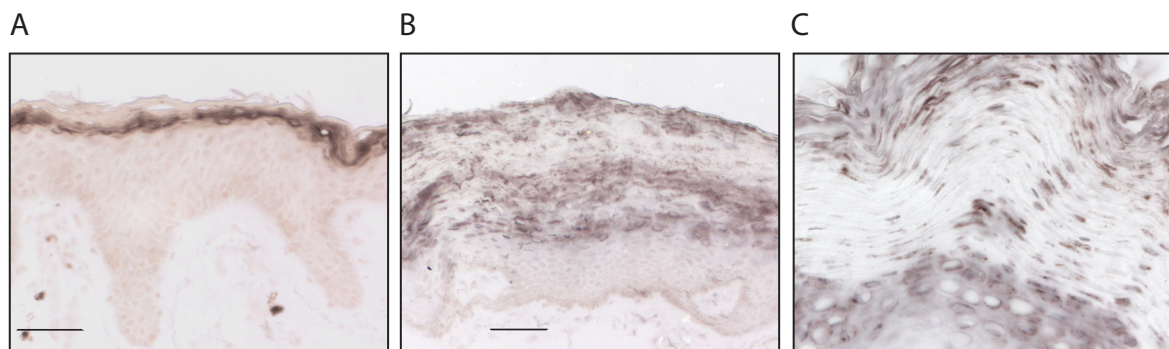

Supplement: Figure S1 — Immunohistochemical detection of profilaggrin in human skin. Epidermal sections from an healthy control (A) and patients with KLICK syndrome (B, C) were stained with antibodies against profilaggrin (Abcam). (C) Magnification of the cornified cell layer of patient epidermis. Bar: 50 µm. (PDF) [file pone.0029471.s001.pdf]

# Supporting Information: Figure S2.

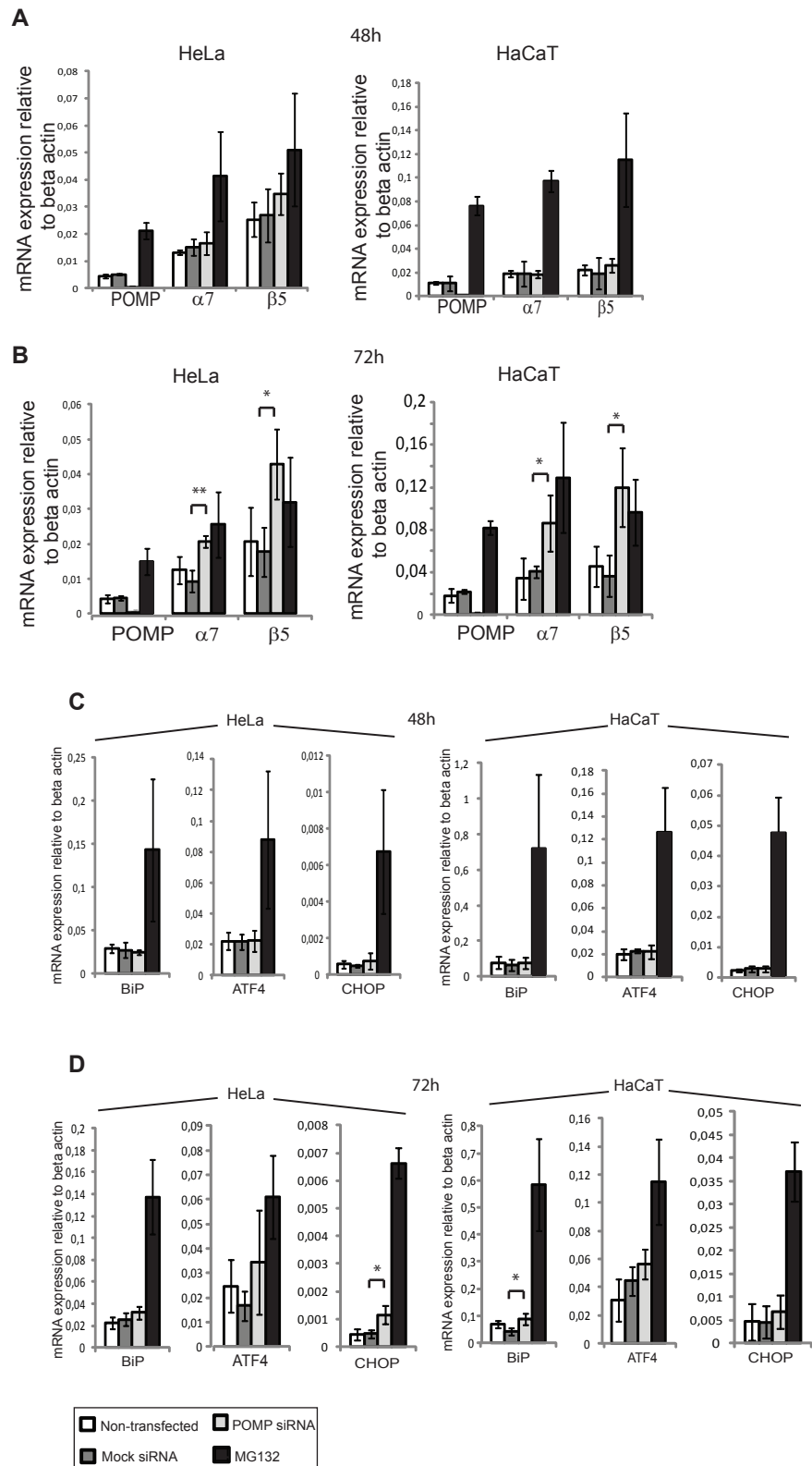

Supplement: Figure S2 — mRNA analysis of POMP siRNA transfected cell lines. POMP was silenced in HeLa and HaCaT cells by siRNA transfection and cells transfected with mock siRNA and cells without transfection (+/−1 µM MG132) were used for comparison. mRNA levels of POMP, α7, β5 (A–B), BiP, ATF4 and CHOP (C–D) were analyzed by qPCR at 48 h (A, C) and 72 h (B, D) post transfection. Beta actin was used as internal control. Differences between POMP siRNA and mock siRNA transfected cells were analyzed using Student's t-test; * = p<0.05, *** = p<0.001. (PDF) [file pone.0029471.s002.pdf]
